# A Research Agenda for Malaria Eradication: Basic Science and Enabling Technologies

**DOI:** 10.1371/journal.pmed.1000399

**Published:** 2011-01-25

**Authors:** The malERA Consultative Group on Basic Science and Enabling Technologies

## Abstract

Today's malaria control efforts are limited by our incomplete understanding of the biology of *Plasmodium* and of the complex relationships between human populations and the multiple species of mosquito and parasite. Research priorities include the development of in vitro culture systems for the complete life cycle of *P. falciparum* and *P. vivax* and the development of an appropriate liver culture system to study hepatic stages. In addition, genetic technologies for the manipulation of *Plasmodium* need to be improved, the entire parasite metabolome needs to be characterized to identify new druggable targets, and improved information systems for monitoring the changes in epidemiology, pathology, and host-parasite-vector interactions as a result of intensified control need to be established to bridge the gap between bench, preclinical, clinical, and population-based sciences.

## Abstract 2

The Malaria Eradication Research Agenda (malERA) consultative group on Basic Science and Enabling Technologies present a research and development agenda for basic research required for malaria eradication.

**Summary Points**

- Creative and collaborative multidisciplinary basic science approaches are needed to address pressing questions about the biology of *Plasmodium*
- The research paradigm needs to shift from a focus on solely parasite or host to one that incorporates the triad of parasite, mosquito, and human host and their respective interactions
- Key research priorities include: the development of in vitro culture systems for all life stages of *P. falciparum* and *P. vivax*, in particular, hepatic stages; improved genetic technologies for the manipulation of *Plasmodium*; and systems-based approaches incorporating cutting-edge technologies such as metabolomics
- Parasite, human, and vector research needs to be backed up by in-depth population-based field studies
- Most importantly, eradication of malaria will require bridging of the artificial gap between bench-based research, preclinical research, clinical research, and population-based science

## Introduction

The current malaria control effort has focused on developing existing products and procedures (for example, drugs and the distribution of bednets) to reduce malaria morbidity and mortality. However, it is commonly accepted that eradication will not be achieved with current tools. Thus, we must now accelerate the development of a new generation of tools and knowledge aimed specifically at malaria eradication. As we look towards this ambitious goal, we must recognize that cutting-edge basic science, novel research strategies, and creative multidisciplinary approaches all need to be mobilized to bridge the gap between bench, preclinical, clinical, and population-based sciences. The malaria science community is now at a turning point where major advances are needed to move the field forward from control towards the goal of global malaria eradication.

The malERA Consultative Group on Basic Science and Enabling Technologies was convened to identify the major knowledge gaps in basic science and to prioritize basic/fundamental science approaches that might have an impact on malaria eradication, particularly with respect to the design of vaccines, drugs, and diagnostics. We recognize that there are likely to be many more research questions that merit equal importance in the broad field of malaria biology than we can cover in this paper, but herein we highlight only those approaches that were discussed by the consultative group and that may have a direct bearing on malaria eradication.

Leading the charge are new molecular, chemical, immunological, and epidemiological research tools that, whilst requiring adaptation to malaria, have realizable rewards in the near future. In particular, developments in systems biology, metabolomics, glycomics, and lipid metabolism and new high-throughput approaches involving chemical biology are likely to be of great use in the field of malaria eradication. From these new avenues of investigation, it is reasonable to expect advances in vaccine development and the identification of novel drug targets. Moreover, the interlacing of high-throughput molecular technologies with population studies will greatly facilitate the rational application of interventions in diverse malaria-endemic environments and, we anticipate, will significantly increase our ability to shed light on complex and heterogeneous host-parasite-vector interactions.

At the basic science level, we specifically identified a deeper understanding of the whole parasitic life cycle and the interaction of the parasite with human and vector hosts at different stages as a high priority. Such knowledge will augment our ability to evaluate current and future interventions and allow us to determine the potential action of drugs or vaccines across all parasite developmental stages. In addition, a life cycle–based perspective will provide insights into the transitions from one host to another and highlight key points, triggers, decisions, and co-incident events as the parasite moves from one life stage to the next that could prove crucial in malaria eradication attempts.

Most importantly, the consultative group recognized that multidisciplinary approaches will be required to exploit and apply new knowledge and techniques in order to make significant and novel gains in combating malaria. The malaria community needs to involve experts who can bring technologies from other seemingly distant areas of basic and applied research such as physics, electronics, information technology, and engineering. By defining desired outcomes, free from the constraints of preconceived ideas based on current tools, an expansion of the malaria research community skill base will create opportunities for lateral thinking and bring with it new approaches not previously considered.

This paper considers the key research priorities identified by the malERA Consultative Group for basic sciences and enabling technologies. Some of these key priorities have also been identified and discussed by the malERA Consultative Groups on vaccines, drugs, and diagnoses and diagnostics [1]–[3].

## *Plasmodium* In Vitro Culture Systems

The development of *Plasmodium* in vitro culture systems that encompass the entire parasite life cycle of *P. falciparum* and *P. vivax* is critical for efforts to develop new vaccines, drugs, diagnostic tests, and challenge/test systems for clinical trials. The development of such systems will require a sustained community-wide collaborative effort and a long-term commitment. Specific stages of the life cycle for human malaria parasites that remain key priorities for in vitro culture development are sporogony, sustained blood-stage culture for *P. vivax*, and the pre-erythrocytic liver stage.

### In Vitro Culture of Mosquito Stages

To date, in vitro culture of mosquito stage parasites and, in particular, in vitro development of sporozoites (sporogonic development) has only been achieved for rodent malaria parasite species; the reproduction of these achievements for *P. falciparum*—and even more so *P. vivax—*has met with limited success [4]–[10]. The need for an effective, in vitro sporogonic culture system is highlighted by the following example. In Brazil, the major local vector *Anopheles darlingi* cannot be reared in the lab and, as in other malaria endemic countries, the importation of laboratory colonies of nonindigenous vector species is understandably prohibited. Such a situation makes it difficult for endemic country scientists to address one of the key issues pertaining to eradication—parasite transmission through the mosquito. Moreover, despite several attempts in the past, an appropriate *Anopheles* midgut cell line model does not exist, which prevents an intimate analysis of *Plasmodium* ookinete invasion of this mosquito tissue [11]. Thus, to facilitate malaria control efforts in Brazil (and in other endemic areas), it is essential that researchers work towards the development of a simple, widely utilizable, and robust mosquito-free (axenic) sporogonic culture system and an in vitro midgut cell invasion assay for the major human malaria parasites.

### *P. vivax* Blood-Stage Cultures

The availability of continuous in vitro blood-stage culture of *P. falciparum* has revolutionized our understanding of the parasite [12], but there is no analogous culture system for *P. vivax* research. In part, this is because *P. vivax* has a predilection for reticulocytes, a cell type that may need to be purified or generated from hematopoietic stem cell progenitor cells—a process that has met with only limited success [13]–[19]. The development of an efficient inexpensive, automated *P. vivax* blood-stage culture system would undoubtedly enhance the study of this parasite's biology. It would also enable in vitro drug susceptibility testing, the development of growth inhibitory assays to test humoral immunity, and, ultimately, the development of new genetic research methods. Importantly, both the asexual and sexual stages would become available for study, which would facilitate the generation of the other stages of the life cycle using defined parasite strains, without the requirement for primates.

### Liver-Stage Cultures

Our current understanding of the biology of the parasite's liver stage (the hypnozoite stage) suggests this stage will be an important target in efforts to eradicate malaria [20]. Specifically, hepatic development occupies a critical position in mediating the establishment of blood-stage infection and, consequently, the transmission of malaria. Moreover, in the case of *P. vivax*, the dormant hypnozoite stages remain in the liver for a variable and protracted period before leading to relapse. Clearly, eradication of *P. vivax* (and *P. ovale*) is unlikely to be attained without developing effective hypnozoiticides.

The availability of a *Plasmodium* liver-stage model would allow the investigation of the host factors that are involved in primary and latent intrahepatic development and of the metabolic pathways that regulate development of this parasitic stage. In addition, the existence of such a model would allow the development of much needed drug screens for this stage that could, like the recently available drug screens for asexual blood-stage infections [21]–[27], take advantage of the unprecedented access to the three chemical compound libraries—GlaxoSmithKline's Tres Cantos Antimalarial TCAMS dataset [24], the Novartis-GNF Malaria Box Dataset, and the St. Jude Children's Hospital Malaria dataset [25]—that are hosted at ChEMBL-NTD (www.ebi.ac.uk/chemblntd), an Open Access repository of primary screening and medicinal chemistry data.

Finally, with the resurgence in interest in genetically attenuated or irradiated sporozoite-based, pre-erythrocytic vaccines [28],[29], a liver-stage model would permit investigation of the molecular basis of their developmental arrest—an understanding that will be critical in both the licensing of such vaccines and in ensuring that breakthrough infections do not arise.

Thus, the development of in vitro systems to understand hypnozoite biology as it relates to liver-stage biology is a clear priority. However, the culture of parasites through the liver stage is likely to be a significant challenge given the intractability of this stage relative to other life stages. Such an endeavour will require a highly collaborative and interdisciplinary approach that includes specialists in the fields of hepatocyte and stem cell biology as well as biomedical engineering. The development of hepatocytes that maintain their polarity and normal trafficking properties is a necessary step towards this kind of model, as is development of primary or immortalized hepatocyte cultures with sufficient life span to allow hypnozoite formation and survival [30]–[34]. Cell lines that allow high infectivity and that can yield high parasite numbers would be especially valuable for generating more useful quantities of parasite material with which to work. Moreover, a single hepatocyte line may not be amenable or useful to all the different subdisciplines present in the malaria community. Some may be appropriate for immunological studies, while others may be suited to drug studies against primary or relapse infection from hypnozoites [1],[2]. Finally, it should be noted that the possibility of using humanized mouse models engrafted with functional human cells and tissues, including human hepatocytes or human hematolymphoid cells, presents a unique in vivo approach that could also facilitate our understanding of *Plasmodium* liver-stage biology [35].

## Primate Models of Disease

Not every aspect of parasite biology can be studied using in vitro culture. In some cases, whole animal models will be needed. For example, validated biomarkers for intrahepatic development and markers of past infection that could help distinguish between new infection and relapse will be important during elimination and can only be identified in whole animal models [3],[36]. Data from primate studies could provide an interim platform for developing novel diagnostics that could inform future work in parallel with in vitro models [37]–[39]. Mechanisms to support cross-institute/laboratory collaborations and access to the few centres with expertise and resources in primate/malaria research would facilitate and enhance a wide range of essential research.

## Development of Genetic Tools for *P. vivax* and Approaches for Systematic Mutagenesis in *Plasmodium*

Major advances towards understanding fundamental aspects of model organisms inherently follow technological innovations that move fields in new directions. Thus, the ability to manipulate the genomes of different *Plasmodium* species has revolutionized malaria research. Nevertheless, we are still a long way from the systematic use of reverse genetics seen in other model systems such as yeast. For example, although the *P. falciparum* genome was completed more than 5 years ago, as many as half of the annotated genes are still listed as having a hypothetical or unknown function; around 90% of the genes have little biological evidence for function. Furthermore, little is being done currently to coordinate the study of individual genes or gene families, with the exception of recent efforts to systematically define the function of proteins involved in erythrocyte remodeling and export [40].

Despite many recent improvements to genetic technologies in *Plasmodium,* many roadblocks that prevent scale-up of genetic manipulation and functional analysis of essential genes need to be overcome [41]–[44]. These roadblocks include the low frequency of homologous recombination in *Plasmodium*, difficulties associated with the manipulation of large fragments of AT-rich and repetitive genomic DNA, and the lack of robust and scalable systems for conditional gene expression. In addition, there are no practical strategies for achieving saturation mutagenesis. Technologies to tackle some of these roadblocks are available for other organisms [45],[46] and need to be introduced to the malaria research agenda.

If these technical limitations can be overcome, systematic mutagenesis on a genome-wide scale will allow us to distinguish essential from redundant metabolic pathways and will be critical to obtaining a comprehensive picture of the stage-specific biology of the parasite that could be targeted with drugs or vaccines. Stable, conditional knock-out approaches for genes that are essential in one life stage but not in another would also identify potential drug targets. Improved genetic technologies will also enable the systematic production of large-scale repositories of gene knock-out or epitope-tagged versions for every plasmodial gene. Such community resources would avoid duplication and benefit from the economy of scale. More importantly, easy access to large numbers of mutants would inspire new experimental approaches, as they have in the yeast field [47]–[49], and widen access to genetic technology.

Finally, the recent completion of several parasite and mosquito genomes [50]–[54] and new insights into the contribution of human and mosquito host genotype to transmission have radically changed how researchers approach malaria. This information, together with an internationally accessible repository of transgenic lines for every *Plasmodium* gene, will change the way that the research community approaches the most basic and relevant questions related to *Plasmodium* biology (of all species) and interactions of the various *Plasmodium* species with their hosts.

## Metabolomics

As with genomic innovations, new technological platforms that permit the deep characterization of the metabolome (complete set of small-molecule metabolites) of *Plasmodium* will identify new potentially druggable targets [55],[56]. Indeed, analysis of the parasite's metabolome is already revealing profound new insights into parasite biology that were not amenable to or that were missed by genomic approaches [57]–[59]. For metabolites that are readily identifiable, differences among parasite strains, under varying drug conditions, or in mutant backgrounds will enhance understanding of the known metabolic pathways present in *Plasmodium* spp. However, many of the measurable compounds are likely to derive from previously undetected novel metabolites (including the products of poorly understood lipid and carbohydrate metabolism). The identification of these compounds could yield key insights for the development of new antimalarial drugs or the control of drug resistance. Moreover, the identification of the metabolic similarities between different parasite stages could provide new approaches to the development of drugs with potential to kill the parasites at many points in their life cycle, possibly in both the human host and the mosquito vector [58]–[61].

Metabolomic approaches should also enable identification of metabolic differences between, for example, patients who are asymptomatic and those with advanced stage cerebral malaria (or other severe syndromes). Metabolomic studies of such samples may not only provide information about the state of the host, but also about the interaction between the host and the parasite. The Consultative Group felt that such studies, which bring together bench scientists and field clinicians, should be encouraged as the true picture of the diversity of metabolic effects can only be fully appreciated from field-derived samples. Finally, the group noted that the application of metabolomic technology will be particularly powerful in unraveling the biochemical strategies of parasites with no or poor genomic resources such as *P. ovale* or *P. malariae*.

## The Importance of Relating Molecular Science to Field Science

The emergence of artemisinin resistance [62],[63] and changes in the interrelationships of humans, mosquitoes, and parasites as elimination proceeds will produce unexpected new challenges. The Consultative Group, therefore, considered it a priority to establish information systems for monitoring the changes in epidemiology, pathology, and host-parasite-vector interactions that result from intensified control and burgeoning elimination efforts so that basic research can react in a timely manner to changing circumstances (see also [36],[64]).

Indeed, a core theme in our discussions was that, throughout the eradication era, basic science and multidisciplinary approaches must be seen as integral components of a Malaria Eradication Research Agenda that are valuable even in the absence of clear field application because one can never predict the impact of novel insights. Enabling technologies cut across many themes in this agenda (see Box 1), and the application of basic science in the field is especially important. For example, genomic, proteomic, and high-throughput immunological methods can now be applied to population studies, greatly increasing their ability to shed light on complex host-parasite-vector interactions [65],[66].

**Box 1. Enabling Technologies: Cross-Cutting Themes**

- Collaborative approaches that bridge the gap between basic laboratory, preclinical, and clinical/population-based sciences
- Complete *P. falciparum* and *P. vivax* in vitro culture systems for the discovery of new targets, the characterization of the entire metabolome, and the evaluation of current and next generation interventions
- Development and wide distribution of several viable, easy to maintain polarized hepatocyte cell lines that support enhanced (>2% infection) *P. falciparum* and *P. vivax* intrahepatic development, and that are amenable to metabolomic, proteomic, glycomic and immunological studies, and the evaluation of new interventions
- Scalable genetic technologies that enable a shared resource containing genome-wide sets of genetically modified (knock-out/tagged) parasite lines (for *P. falciparum, P. vivax,* and the murine malaria parasites) to be maintained
- Novel classes of molecules that can function as chemical tools for probing the function of genes at transition stages; most especially the commitment to dormant liver stages and gametocytogenesis

## Human Host Factors and Improving Epidemiological Models

No campaign for the control or elimination of malaria can proceed without a detailed appreciation of the epidemiology of the disease and of host-parasite-vector interactions. As increasing parts of the world move towards elimination, a deeper understanding of the basic science of host-parasite-vector population interactions in disease transmission and of the changes in these interactions that result from intensified control and elimination efforts will be increasingly important [64].

For example, mixed species and strain infections are common in natural malaria infections in both human and vector hosts [67],[68]. Application of next generation high-throughput sequencing and genotyping of mixed infections in both obligate hosts will help to identify important target genes and phenotypes and will provide insights into whether and how parasites impact each other's behaviour in the context of the human host and transmission through the vector host that will be important as elimination proceeds.

Similarly, a better understanding of the human response to malaria will be increasingly important as elimination proceeds. Despite many decades of studies on immune responses to malaria, there is still no consensus on an immune correlate of protection [69],[70]. Well designed, longitudinal studies in which the exposure to malaria and protection against uncomplicated or severe malaria are reliably assessed are required to remedy this shortcoming. Other modern technologies that offer new approaches to understanding the potential mechanism of action of compounds or antibodies on malaria will also need to be fully introduced into ongoing and planned longitudinal studies of human populations.

## Vector-Host-Parasite Interactions

Strategies aimed at decreasing mosquito life span are predicted to impact upon transmission (see also [2],[71]). Research that investigates the parasite stages that develop within the mosquito and their transmission through the vector is likely to be of great use, therefore, in malaria control and eradication. Focused research efforts designed to understand the epidemiology of the gametocyte and how it varies with species, with host, and with the environment are required. Insights arising from such research will be critical for determining the driving factors for new human and mosquito infections, and manipulation of these factors will open up new avenues for targeting the key parasite regulatory switches that occur when a parasite undergoes a transition event. Importantly, however, such a focus on transmission need not necessarily be aimed at finding a magic bullet—a compound that can work against all parasite stages in all hosts. Instead, there will be significant utility in developing several inhibitors with similar pharmacokinetic and pharmacodynamic profiles that affect different metabolic pathways and stages in a combined drug treatment regimen. For now, the absence of compounds that preferentially affect gametocytogenesis, gamete-ookinete, or ookinete-oocyst transition as well as the lack of understanding of the mechanism of action for those very few currently available compounds highlights the need for renewed efforts in this area [72].

## Concluding Remarks

From our discussions, we propose a basic science research and development agenda for malaria eradication (Box 2) that will hopefully yield new interventions that are not hindered by the current drug resistance status of the parasites or by changes in environmental and host factors.

**Box 2. Summary of the Research and Development Agenda for Basic Science Research**

- A research paradigm shift away from the “parasite-first” approach to an examination of what the human and mosquito host cells provide to the developing parasite is needed to complement on-going approaches
- A new approach is needed to support collaborative and truly cross-disciplinary arrangements among scientists to bridge the gap between basic laboratory and clinical/population-based sciences and to meet the scientific benchmarks outlined by malERA
- Desired target product profiles need to be defined without preferred technological approaches being suggested to create opportunities for lateral thinking by experts bringing new approaches from different fields
- Careful evaluation and appropriate use of today's technologies from the physical, chemical, and biomedical engineering sciences is needed to improve the molecular understanding of parasite developmental biology and of the mammalian host-parasite-vector interactions
- Mechanism of action studies for drugs and vaccines in the current pipeline are also needed to inform future strategies for the development of the next generation of interventions and therapeutics
- The study of human host and vector factors in large-scale, long-term population-based field studies and the use of appropriate technologies in translation applications is also essential.

Central to this agenda is our contention that the establishment of a spectrum of creative and novel eradication interventions will require a strong commitment to collaborative work, wherein interdisciplinary teams of basic scientists, both within and outside of the malaria field, are organized and tasked with achieving well-defined research milestones. It is crucial that scientists have the possibility and flexibility to move between the field and the bench for collaborative translational research, an emerging specialty in its own right. Once individuals embrace the diversity of expertise necessary in the malaria research of the future, the promotion of a greater collaborative culture will inevitably make translation from bench to bedside more readily achievable. However, the 10–15-year timeline of translation from the bench to practical use in the clinic or field remains a significant barrier to progress that has to be recognized. Finally and importantly, our challenge to basic and applied scientists to engage in stronger partnership across projects and disciplines overrides some of the current guiding principles in science such as institutional and individual performance assessments and impact factors. These criteria may have helped to shape individual careers but they have rarely helped to answer major public health questions and should not be allowed to interfere with progress towards malaria eradication.
