# Correction: A Research Agenda for Malaria Eradication: Basic Science and Enabling Technologies

**DOI:** 10.1371/annotation/e6a85761-7a23-435a-97df-f13c1ba4e8da

**Published:** 2011-05-11

**Authors:** The malERA Consultative Group on Basic Science and Enabling Technologies

Tim Gilberger should be included in the list of participants in the malERA Young Investigators/Basic science meeting 8-9 October 2009, Boston, Massachusetts, USA. He is affiliated with the Bernhard Nocht Institute for Tropical Medicine, Hamburg, Germany.

